# Adiponectin is associated with poor prognosis in carcinoma patients: evidence from a meta-analysis

**DOI:** 10.1186/s12944-015-0157-4

**Published:** 2015-11-26

**Authors:** Jiaxiang Ye, Zhongguo Liang, Qian Liang, Jinyan Zhang, Sufei Mao, Rui Liang

**Affiliations:** Department of Medical Oncology, The Cancer Institute, Affiliated Tumor Hospital of Guangxi Medical University, No. 71, Hedi Road, Nanning, Guangxi 530021 P.R. China; Graduate School of Guangxi Medical University, Nanning, Guangxi 530021 P.R. China; Department of Anesthesiology, The Cancer Institute, Affiliated Tumor Hospital of Guangxi Medical University, No. 71, Hedi Road, Nanning, Guangxi 530021 P.R. China

**Keywords:** Adiponectin, Prognosis, Cancer, Meta-analysis

## Abstract

**Background:**

Studies have come to conflicting conclusions about whether adiponectin (APN) expression is associated with cancer prognosis. To help resolve this question, we meta-analyzed the available evidence.

**Methods:**

PubMed, EMBASE, the Cochrane Library, the Chinese Biological Medical Database and the Chinese National Knowledge Infrastructure Database were systematically searched to identify all eligible studies examining APN expression and prognosis for patients with any type of cancer. Pooled hazard ratios (HRs) and corresponding 95 % confidence intervals (CIs) related to overall survival (OS) or disease-free survival (DFS) were calculated.

**Results:**

Ten studies involving 999 patients were meta-analyzed. Analysis across all patients revealed no significant association between high/positive APN expression and DFS, but they did show a significant association between high/positive APN expression and OS (HR 1.51, 95 %CI 1.21 to 1.89). Subgroup analysis showed that high/positive APN expression in non-Asians was significantly associated with both DFS (HR 1.36, 95 % CI 1.03 to 1.80) and OS (HR 1.53, 95 %CI 1.20 to 1.96), but no such associations were observed in Asians. In addition, high/positive APN expression was significantly associated with OS across all patients with hepatocellular carcinoma (HR 1.89, 95 %CI 1.20 to 2.98).

**Conclusions:**

The available evidence suggests that high/positive APN expression is associated with poor prognosis for patients with various carcinomas, especially for non-Asian cancer patients and for all patients with hepatocellular carcinoma. These findings should be confirmed and extended in large, well-designed studies.

## Background

Cancer remains a frequent cause of death worldwide, even though diagnostic and operative techniques have improved dramatically. In 2008, approximately 12.7,000 000 people were diagnosed with cancer around the world, and 7.6,000 000 people died from cancer-related causes [1]. This highlights the continuing importance of identifying prognostic factors that can better guide survival prediction as well as treatment and management strategies.

Large epidemiological studies have shown a significant association of obesity with various carcinomas, including breast, colorectal, renal, endometrial, pancreatic, esophageal, and biliary [2–4]. This implies that the prevalence of obesity, in part, which has been rising in parallel with living standards in developed or developing countries, will contribute to increasing incidence rates of many cancers.

One link between obesity and cancer may be adiponectin (APN), also called gelatin-binding protein 28, which is the most abundant of several adipokines secreted primarily by adipose tissue [5]. APN may play a major role in cancer, and several studies suggest it may also be a prognostic factor for cancer patients, but results from prognostic studies have often been contradictory. For example, studies have shown that increased APN may be associated with poor survival in patients with hepatocellular carcinoma (HCC) [6, 7], whereas a third study found high APN expression to be associated with favorable prognosis in such patients [8]. One study reported that elevated APN levels were associated with reduced disease-free survival (DFS) of patients with breast cancer [9], while another failed to find any significant association between APN expression and prognosis of such patients [10]. Other studies have similarly failed to find a significant relationship between APN expression and survival in patients with lung or gastric cancer [11, 12]. One study reported an association between high APN levels and poor survival in patients with childhood non-Hodgkin’s lymphoma [13].

To address more comprehensively the question of whether APN expression is associated with cancer prognosis, and to examine whether this association depends on patient or cancer characteristics, we systematically searched the research literature and meta-analyzed available evidence. As far as we know, this is the first reported meta-analysis of the association between APN expression and cancer prognosis.

## Patients and methods

### Literature searching

PubMed, EMBASE, the Cochrane Library, the Chinese Biological Medical (CBM) database and the Chinese National Knowledge Infrastructure (CNKI) database were systematically searched to identify studies published through February 30, 2015 that examined the association between APN expression and cancer prognosis. Searches were carried out without restrictions on publication language using various combinations of customized terms and the MeSH-indexed terms “adiponectin”, “prognosis”, “outcome”, “survival”, and “cancer”. The following sequential search strategy was applied for each database: (#1) ‘adiponectin’: ab, ti OR ‘APN’: ab, ti OR ‘adiponectin’/exp; (#2) ‘survival’: ab, ti OR ‘prognosis’: ab, ti OR ‘prognostic’: ab, ti OR ‘outcome’: ab, ti OR ‘prognosis’/exp OR ‘treatment outcome’/exp; (#3) ‘neoplasm’: ab, ti OR ‘cancer’: ab, ti OR ‘carcinoma’: ab, ti OR ‘tumor’: ab, ti OR ‘neoplasm’/exp OR ‘carcinoma’/exp; (#4) #1 AND #2 AND #3, but search strings were adjusted accordingly for the other databases. Reference lists in identified articles were searched manually to identify additional studies.

### Study inclusion and exclusion

Inclusion and exclusion criteria were established before searching the literature. To be included in our meta-analysis, studies had to (1) investigate the correlation between APN expression and prognosis of cancer patients, and (2) provide sufficient information to obtain the hazard ratio (HR) and 95 % confidence intervals (CIs) related to overall survival (OS) or DFS.

Reviews, abstracts submitted to a conference, letters to the editor, case reports and comments were excluded. When studies reported on the same or overlapping patient populations, only the study with the most complete data set and most rigorous methodology was used.

### Data extraction

Two investigators (JXY, ZGL) independently extracted the following data from included studies: first author’s name, year of publication, country/region and ethnicity of study population, type of cancer, number of patients, tumor stage, follow-up period, method to detect APN expression, cut-off value for APN positivity, and HRs with corresponding 95%CIs. When HRs could not be directly extracted from original reports, they were extracted from Kaplan-Meier curves as reported by Tierney *et al*. [14]. If the original articles categorized APN expression levels into tertiles, we extracted HRs and 95 %CIs relating the top tertile to the bottom tertile, as described by Danesh *et al*. [15], or we extracted HRs and 95 %CIs from Kaplan-Meier curves. If a study reported HRs and 95 %CIs for both univariate and multivariate analyses, only the results of multivariate analysis were used.

### Quality assessment

The quality of all eligible studies was evaluated using The Quality In Prognosis Studies (QUIPS) tool [16, 17], which bases its assessment on six study dimensions: study participation, study attrition, prognostic factor measurement, outcome measurement, confounder measurement and approaches for accounting and analyzing. For each dimension, an assessment of ‘Yes’ indicates a low risk of bias; ‘Partly’, moderate risk of bias; ‘No’, high risk of bias; and ‘Unsure’, unclear risk of bias. Each study was also assigned an overall assessment of potential bias as low, moderate, or high.

### Statistical analysis

HRs with corresponding 95 %CIs were calculated using RevMan 5.1.0 (The Cochrane Collaboration, Oxford, UK) to assess the correlations between APN expression and cancer prognosis, quantified in terms of OS and DFS. Heterogeneity among studies was assessed using the Q-test and I^2^ statistics. When homogeneity was considered significant (P_heterogeneity_ ≥ 0.1), a fixed-effect model was used; otherwise, a random-effect model was used.

Subgroup analysis was also conducted according to patient ethnicity and cancer type. We planned to aggregate together in a category of “other cancers” any studies that were the only ones in the meta-analysis to cover a given type of cancer.

To assess publication bias, we performed Begg’s test [18] and Egger’s test [19] using Stata 12.0 (Stata Corporation, College Station, TX).

## Results

### Study selection and characteristics

**Fig. 1:**
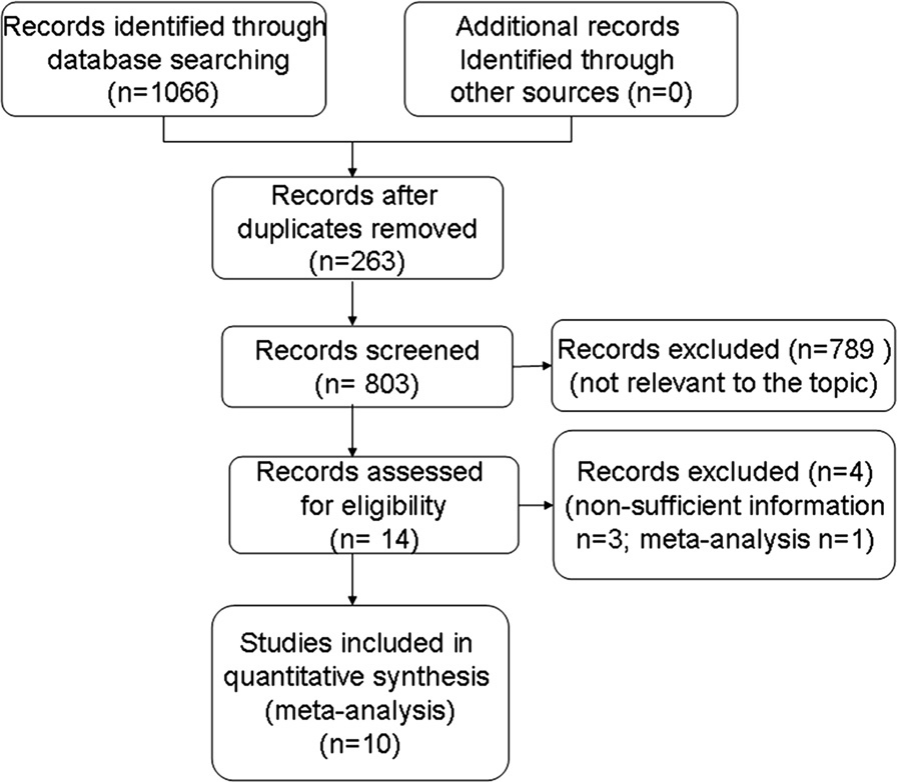
Flow diagram of study selection for the meta-analysis

The search strategy yielded 1066 records, from which 263 duplicate publications were eliminated and 789 were excluded as irrelevant based on a review of titles and abstracts. The remaining 14 studies were read in full to assess eligibility. This led us to exclude three studies because they failed to report sufficient information to estimate HRs and 95 %CIs [20–22], as well as one study because it was a meta-analysis [23]. In the end, 10 studies were included in the meta-analysis [6–13, 24, 25] (Fig. 1, Table 1).

**Table 1:** Main characteristics of the studies included in the meta-analysis examining the relationship between adiponectin expression and survival in patients with various types of cancer

Study	Country	Patient number	Races	St-age	Follow-up(m)	Detected method^a^	Cut-off value	Tumor type	Survival analysis	Source of HR	Analytic method
Wang 2014 [6]	China	85	Asian	I ~ IV	NA	IHC	Low:- ~ + High:++ ~ +++^b^	HCC	OS	SC	U
Siegel 2014 [7]	America	140	Non- Asian	A ~ C^c^	Median 8	RIA	Low:<median (13,050 ng/ml) High:≥median	HCC	OS	Report	M
Cubukcu 2014 [10]	Turkey	38	Non- Asian	I ~ III	Median 30	IHC	Negative: No cells stained Positive:>0 % of cells stained	BC	OS, DFS	Report	M
Canhoroz 2014 [24]	Turkey	53	Non- Asian	II ~ III	Mean 41	IHC	Negative:<5 % of cells stained Positive:≥5 % of cells stained	CRC	OS, DFS	SC	U
Lee 2014 [9]	Korea	247	Asian	I ~ IV	Median 50.4	ELISA	Low:< 8.0 ng/ml High:≥11.8 ng/ml	BC	DFS	Report^d^	M
Shin 2014 [8]	Korea	75	Asian	I ~ IV	Mean 82.5	IHC	Negative: No cells stained Positive:>0 % of cells stained	HCC	OS, DFS	Report (OS); SC (DFS)	M
Kerenidi 2013 [11]	Greece	80	Non- Asian	I ~ IV	NA	RIA	Low:<median (14.39 ng/ml) High:≥median	LC	OS	Report	U
Tsukada 2011 [12]	Japan	100	Asian	I ~ IV	NA	ELISA	Low: < 7 ug/ml High:≥7 ug/ml	GC	OS	SC	U
Petridou 2009 [13]	Greece	121	Non- Asian	I ~ IV	Median 74.3	RIA	Low:<5.94 ug/ml High:≥5.94ug/ml	NHL	DFS, OS	Report	M
Ferroni 2007 [25]	Italy	60	Non- Asian	A ~ C^e^	Median 37	EIA	Low:<6.39 ug/ml High:≥6.39ug/ml	CRC	DFS	SC	U

BC, breast cancer; CRC, colorectal cancer; HCC, hepatocellular carcinoma; GC, gastric carcinoma; LC, lung cancer; NHL, non-Hodgkin’s lymphoma; EIA, enzyme immunoassay; ELISA, enzyme-linked immunosorbent assay; RIA, radioimmunoassay; IHC, immunohistochemistry; DFS, disease-free survival; OS overall survival; SC, survival curves; U, univariate, M, multivariate; HR, hazard ratio; NA, not available

^a^Detected specimens of IHC was tumor tissue, all other specimens were serum

^b^Semiquantitative scoring system

^c^Barcelona Clinic Liver Cancer stage

^d^HRs and 95%CIs of the top Tertile vs. the bottom Tertile

^e^Dukes’ stage

### Study quality

Quality assessment indicated that all 10 included studies involved samples that were likely to represent the key characteristics of the target population of cancer patients, reported data that adequately described the sample, assessed adequately the prognostic factor(s) of interest in study participants and used statistical analysis appropriate to the study design. However, three studies [6, 11, 12] did not report the duration of follow-up, so they received an assessment of “Partly” on the dimension of outcome measurement. In five studies [7, 8, 10, 11, 25], important potential confounders were not matched between cases and controls analyzed. Since three of these studies [7, 8, 10] adequately measured potential confounding variables using prespecified multivariate analysis, they were assessed with “Yes” on the dimension of confounder measurement and accounting; the remaining two studies [11, 25] received an assessment of “No”.

Altogether, six of the 10 studies were judged to have low overall risk of bias, two to have moderate overall risk of bias, and two to have high risk of bias (Table 2).

**Table 2:** Risk of bias for each study based on QUIPS quality assessment

Study	Study partici-pation	Study attrition	Prognostic factor measurement	Outcome measure-ment	Confounding measurement and account	Analysis approaches	Overall appraisal of potential bias
Wang 2014 [6]	Yes	Yes	Yes	Partly	Yes	Yes	Moderate
Siegel 2014 [7]	Yes	Yes	Yes	Yes	Yes	Yes	Low
Cubukcu 2014 [10]	Yes	Yes	Yes	Yes	Yes	Yes	Low
Canhoroz 2014 [24]	Yes	Yes	Yes	Yes	Yes	Yes	Low
Lee 2014 [9]	Yes	Yes	Yes	Yes	Yes	Yes	Low
Shin 2014 [8]	Yes	Yes	Yes	Yes	Yes	Yes	Low
Kerenidi 2013 [11]	Yes	Yes	Yes	Partly	No	Yes	High
Tsukada 2011 [12]	Yes	Yes	Yes	Partly	Yes	Yes	Moderate
Petridou 2009 [13]	Yes	Yes	Yes	Yes	Yes	Yes	Low
Ferroni 2007 [25]	Yes	Yes	Yes	Yes	No	Yes	High

QUIPS = Quality in Prognosis Studies

Yes = a low risk of bias; Partly = moderate risk of bias; No = high risk of bias

### APN expression and OS

**Fig. 2:**
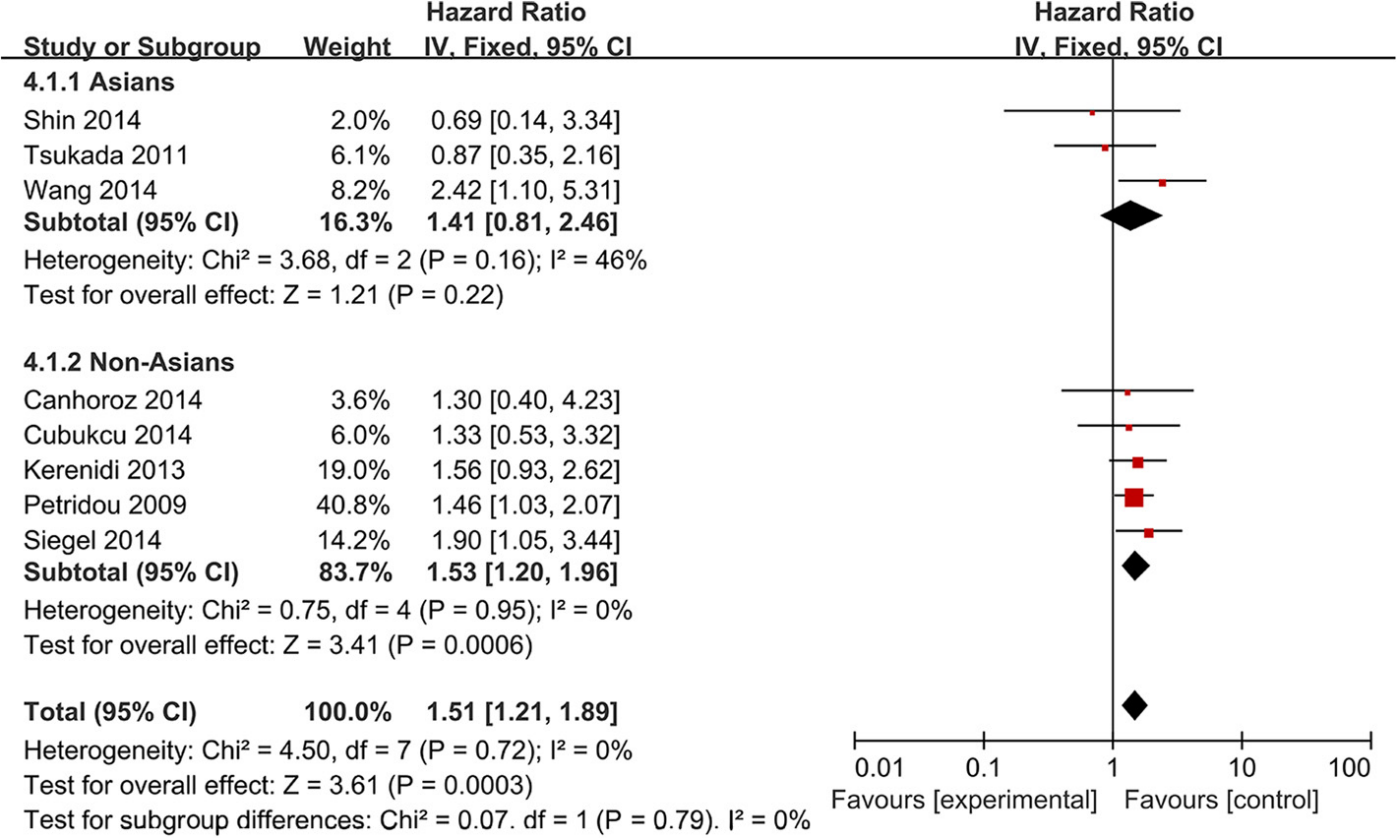
Forest plot of the relationship between APN expression and OS in Asians and non-Asians with a variety of cancers

Pooling data from eight studies [6–8, 10–13, 24] showed that high/positive expression of APN was significantly associated with OS in patients with various carcinomas (HR 1.51, 95 %CI 1.21 to 1.89; Fig. 2).

**Fig. 3:**
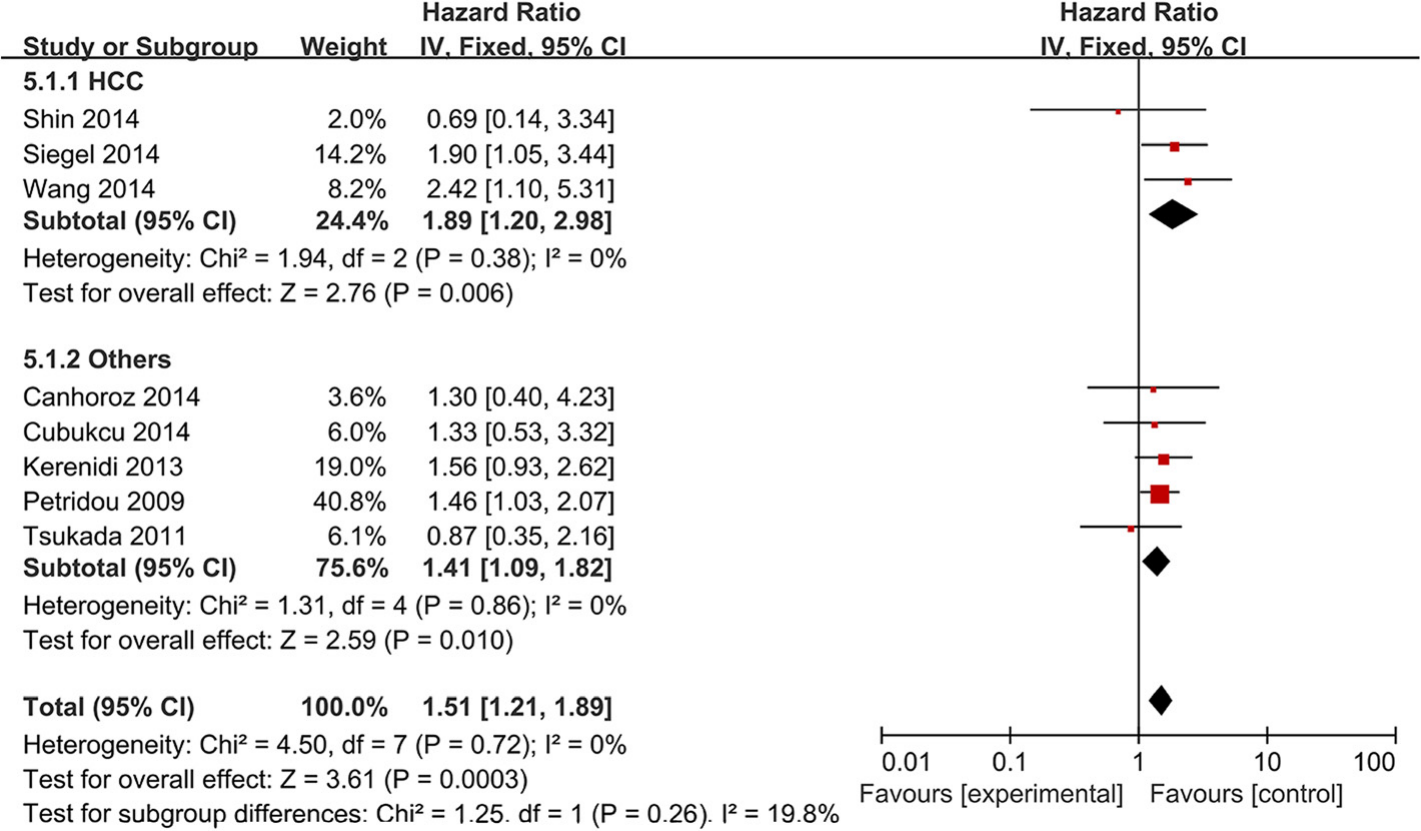
Forest plot of the relationship between APN expression and OS across all patients with different cancers

Subgroup analysis by patient ethnicity and tumor type (Figs. 2 and 3) showed a significant relationship between high/positive APN expression and OS in non-Asian patients (HR 1.53, 95 %CI 1.20 to 1.96), but not in Asian patients (HR 1.41, 95 %CI 0.81 to 2.46). The association between high/positive APN expression and OS was also observed among patients with HCC (HR 1.89, 95 %CI 1.20 to 2.98) and “other cancers”.

### APN expression and DFS

**Fig. 4:**
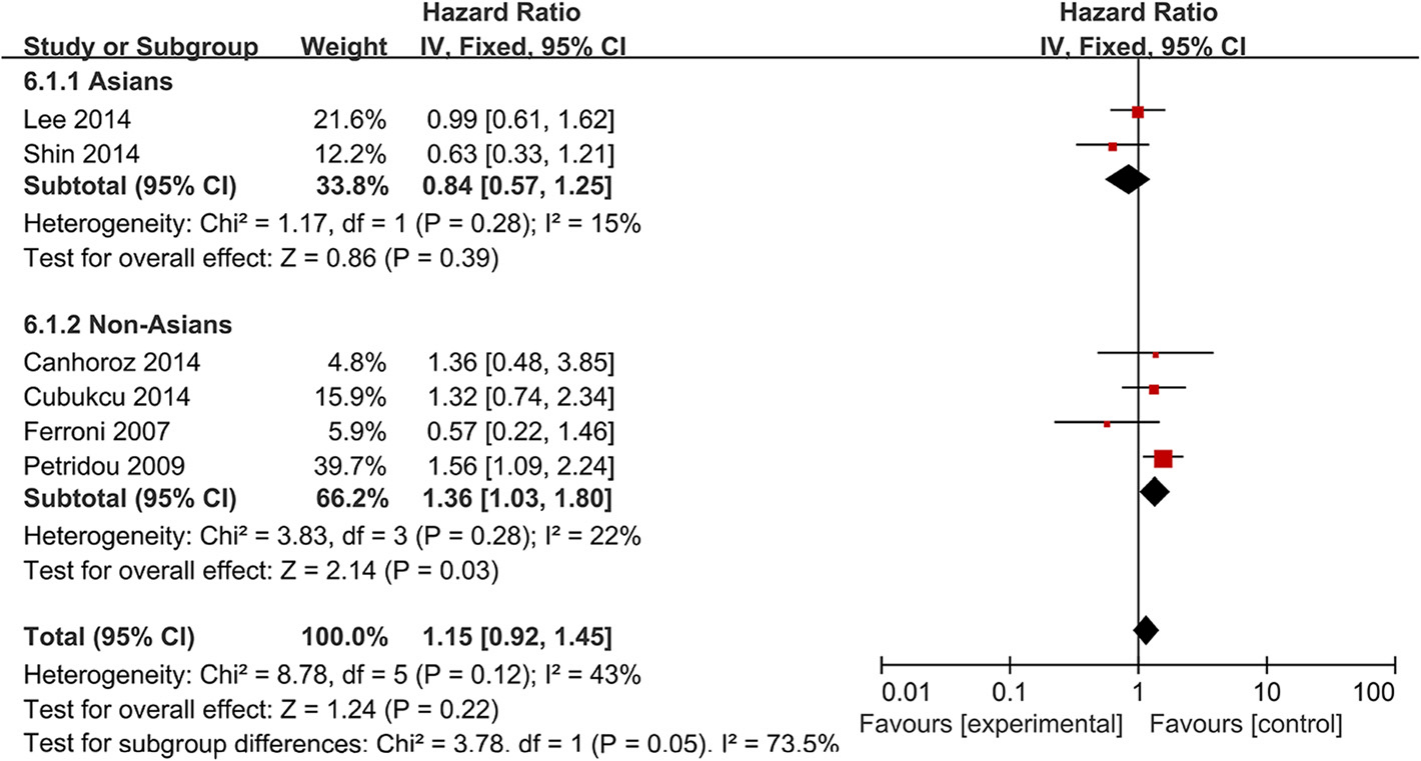
Forest plot of the relationship between APN expression and DFS in Asians and non-Asians with a variety of cancers

**Fig. 5:**
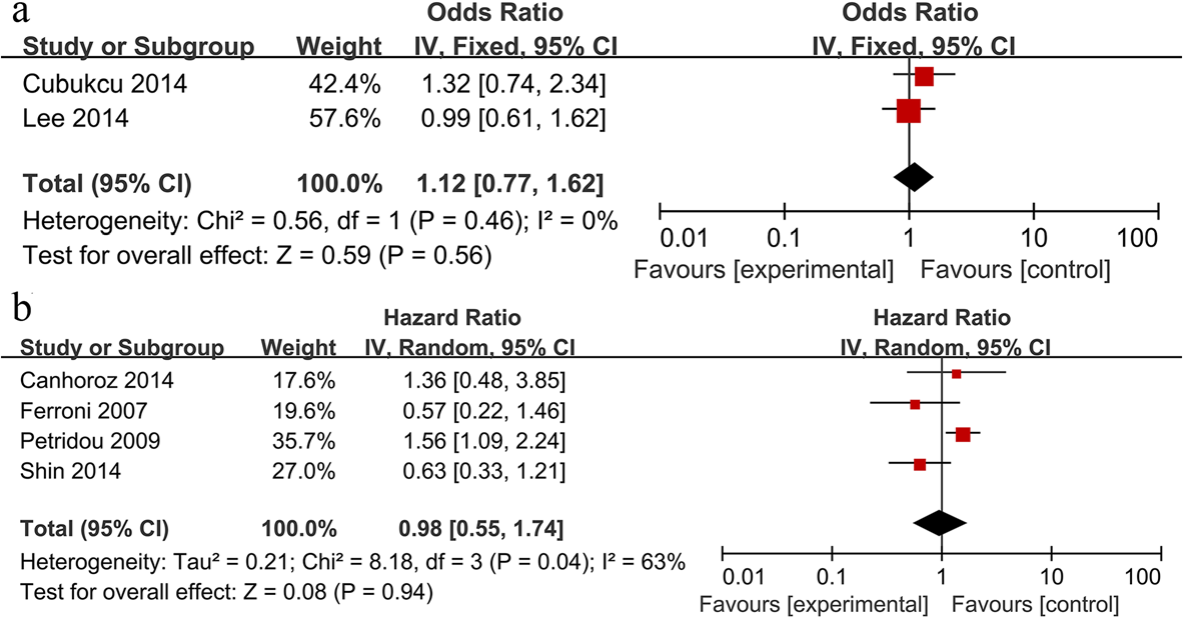
Forest plot of the relationship between APN expression and DFS across all patients with different cancers. **a**. Forest plot of breast cancer in fixed-effect model; **b**. Forest plot of cancers other than breast cancer in random-effect model

Pooling data from six studies [8–10, 13, 24, 25] showed no significant association between high/positive APN expression and DFS in patients with various cancers (HR 1.15, 95 %CI 0.92 to 1.45; Fig. 4). Similar negative results were obtained in subgroup analyses (breast cancer: HR 1.22, 95 %CI 0.77 to 1.62; Figs. 4 and 5), with the exception of analysis by patient ethnicity: in non-Asian cancer patients, high/positive APN expression was significantly associated with DFS (HR 1.36, 95 %CI 1.03 to 1.80).

### Publication bias

Egger’s and Begg’s tests to assess risk of publication bias suggested no significant risk, with the respective tests returning P values of 0.425 and 0.386.

## Discussion

Despite numerous studies examining a possible link between APN expression and cancer prognosis, the evidence remains unclear. Combining the statistical power of 10 studies involving 999 patients, the present meta-analysis suggests that positive/high APN expression is not significantly associated with DFS in various carcinomas but is significantly associated with OS (HR 1.51, 95 %CI 1.21 to 1.89). This suggests that positive/high APN expression may be a useful biomarker to predict poor prognosis in patients with various carcinomas.

Subgroup analyses suggest that the relationship of APN expression with DFS and OS may depend on patient ethnicity. While APN expression was not significantly associated with either DFS or OS among Asian patients with various cancers, it was associated with both outcomes in non-Asian patients (DFS, HR 1.36, 95 %CI 1.03 to 1.80; OS, HR 1.53, 95 %CI 1.20 to 1.96). This differential ethnic effect may reflect differences in genetic background, lifestyle, dietary habits, and environmental factors.

Subgroup analyses further suggest that the relationship of APN expression with DFS and OS may depend on tumor type. Thus, high/positive APN expression had no association with DFS in patients with breast cancer, but correlated with OS in patients with HCC (HR 1.89, 95 %CI 1.20 to 2.98). Unfortunately our meta-analysis was unable to provide a clear answer about a possible association between APN expression and prognosis for other types of cancer because too few studies in our data set of each tumor type. This highlights the need for large, well-controlled studies of APN expression in other cancers.

Our findings that elevated APN expression is associated with poor cancer prognosis are consistent with a meta-analysis of 16 prospective studies involving 14,063 subjects, which showed that high APN level is associated with increased risk of mortality in patients with cardiovascular disease [26]. Prospective analysis of 1000 community-dwelling adults 65 years and older suggested that high APN concentration is significantly associated with increased risk of all-cause and cardiovascular mortality [27].

These findings seem paradoxical given that many studies in vitro and in vivo have shown APN to have significant anti-diabetic, anti-atherosclerotic, anti-inflammatory, anti-proliferative and anti-carcinogenic activity [28]. Several mechanisms have been proposed to explain this so-called “adiponectin paradox”. One possibility is APN resistance: even when APN is abundantly expressed, it may fail to protect against poor prognosis because the APN receptor is down-regulated or the APN signaling pathway is dysfunctional. Indeed, many patients with HCC have liver fibrosis or cirrhosis, both of which have been associated with APN receptor down-regulation in liver tissue and reduced clearance of APN, resulting in a state of APN resistance [29–31]. Another possible mechanism of APN resistance is that APN expression initially increases to compensate for disease progression, but the higher levels of APN turn out to be ineffective because of overall deterioration of the patient’s condition [26].

A second possible explanation of the adiponectin paradox is that APN promotes AKT-mediated activation of cancer cells [5, 6, 32]; such activation is a significant predictor of worse survival [33, 34]. A third possible explanation is that APN promotes angiogenesis in tumors [35–37].

The results of our meta-analysis are consistent with those of an earlier meta-analysis [23] involving 705 HCC patients and 1390 healthy controls, which found significantly higher serum APN levels in HCC patients than in healthy controls. Those authors concluded, paradoxically, that elevated serum APN levels may be associated with slower progression of HCC patients, but they did not support their claim with survival data. The present meta-analysis, in contrast, is based on rigorous assessment of OS and DFS in the literature.

Despite its strengths, our meta-analysis has several limitations. First, the accuracy of the meta-analysis may be affected by the variety of APN cut-off values used to detect expression in the included studies. Second, we extracted several HRs from survival curves or HRs for top and bottom tertiles in the original articles, which may have introduced small errors. Third, though our analyses suggested no significant risk of publication bias, the meta-analysis included a small number of studies in global or subgroup analyses, it might result in little bias of finding. Fourth, the patient populations were heterogeneous because they had different types of cancer. Moreover, different detected specimens (tumor tissue or serum) and detected methods might also increase the risk of bias. Therefore, these findings should be used with caution.

## Conclusions

The available evidence suggests that high/positive APN expression is associated with poor prognosis for patients with various carcinomas, especially for non-Asian cancer patients and for all patients with hepatocellular carcinoma. Future studies should confirm and extend our findings. In particular, large, well-designed studies are needed to examine the possible association of APN expression with prognosis in non-HCC cancers in Asians and non-Asians.
